# Cytomegalovirus retinitis in a patient with secondary acute lymphosarcoma leukemia undergoing allogeneic hematopoietic stem-cell transplantation: A rare case report: a care-compliant article

**DOI:** 10.1097/MD.0000000000006878

**Published:** 2017-05-12

**Authors:** Ning Zhao, Lei Liu, Junjie Xu

**Affiliations:** a Department of Ophthalmology; b Department of Laboratory Medicine, Key Laboratory of AIDS Immunology of National Health and Family Planning Commission, The First Affiliated Hospital of China Medical University, Shenyang, Liaoning, China.

**Keywords:** cytomegalovirus retinitis, fluorescein angiography, immunocompromised, pathology section

## Abstract

**Rationale:**

Cytomegalovirus (CMV) retinitis is a common opportunistic infection in immunocompromised patients, which may lead to blindness. CMV retinitis is not an uncommon infectious disease in patients with immune regulatory abnormalities, for example, human immunodeficiency virus (HIV) patients. However, CMV retinitis in a patient with acute lymphosarcoma leukemia (ALL) undergoing allogeneic hematopoietic stem-cell transplantation (HSCT) phase is very rare.

**Patient concerns:**

A case of CMV retinitis in a patient receiving immunosuppressive therapy as a part of ALL allogeneic HSCT is described including the pathogenesis, clinical signs, and therapy.

**Diagnoses:**

CMV retinitis.

**Interventions:**

Ganciclovir intravitreal injection at weekly intervals for 4 weeks.

**Outcomes:**

Patient's vision had improved and the load of CMV deoxyribonucleic acid (DNA) in the aqueous humor declined. The CMV retinitis and perivascular of retina infiltration regressed.

**Lessons:**

We propose that the concentration of CMV DNA load in the aqueous humor could be useful in making the diagnosis and in selecting the optimal treatment in this kind of CMV retinitis.

## 1 Introduction

Opportunistic infections suffered by immunocompromised patients are usually caused by cytomegalovirus (CMV) that is a deoxyribonucleic acid (DNA) virus.^[1]^ A CMV infection may cause dysfunctions in many organs including the retina. Generally speaking, CMV infection is diagnosed by the isolation of CMV or by the detection of viral protein/nucleic acid in body fluids or specimen tissue. However, CMV retinitis is usually diagnosed by a competent ophthalmologist who detects the presence of lesions during fundoscopy in immunocompromised individuals such as HIV patients.^[2]^ CMV retinitis is very rare in patients undergoing allogeneic hematopoietic stem-cell transplantation (HSCT) during the immunosuppressive phase of this treatment. We performed a literature search for more information about this secondary disease, but currently there are only a few case reports of CMV retinitis occurring in patients receiving allogeneic HSCT.^[3–6]^ Little is known about the pathogenesis or which laboratory tests are useful in the diagnosis of CMV retinitis in patients receiving immunosuppressive therapy as a part of allogeneic HSCT. We present a rare case of this kind of CMV retinitis and describe the laboratory tests applied in its diagnosis.

## 2 Case report

**Figure 1:**
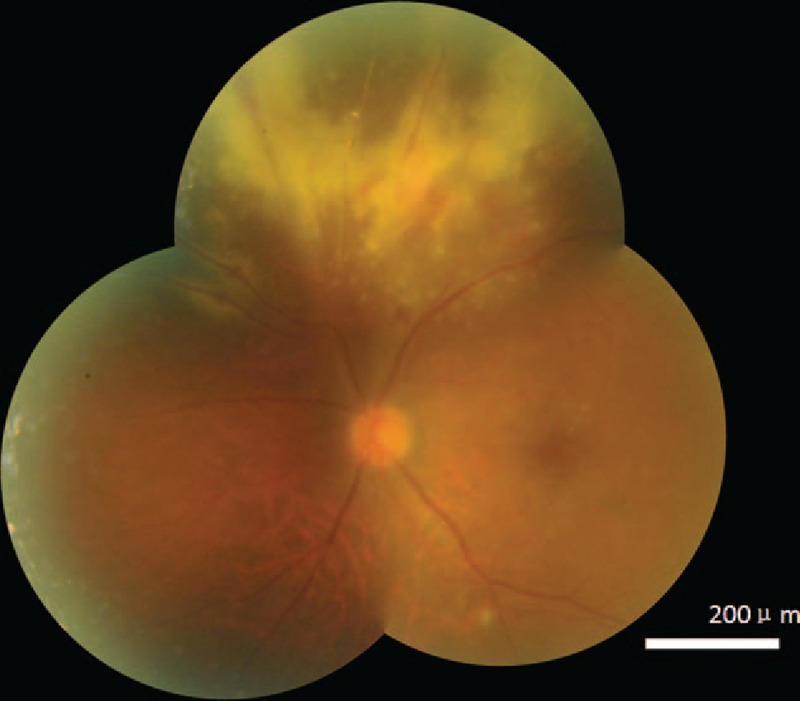
Cytomegalovirus retinitis was characterized by exudates, retinal necrosis, occlusion of retinal veins, and hemorrhage.

**Figure 2:**
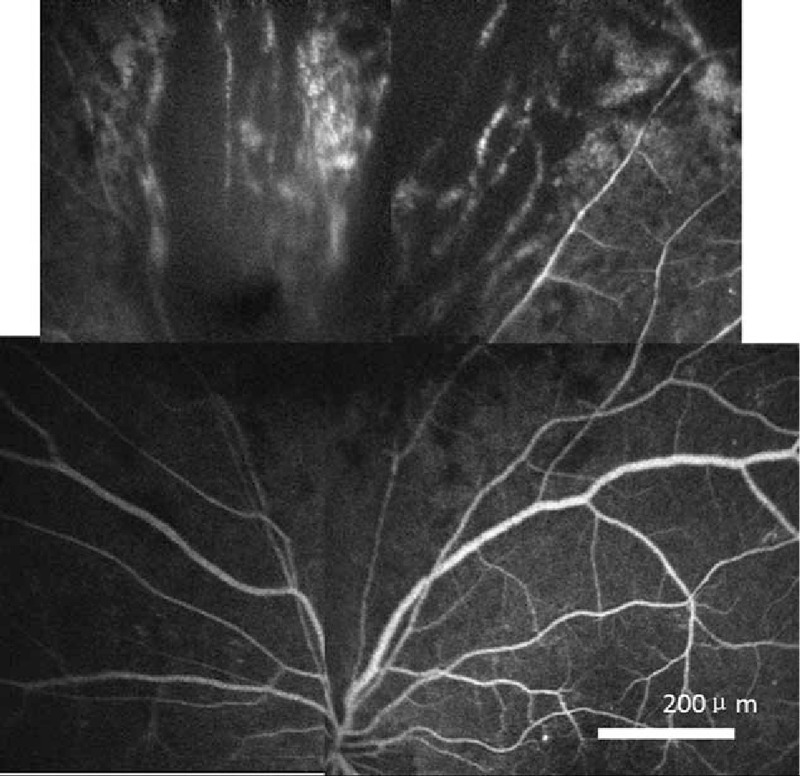
The fluorescein angiography revealed retinal leakage, nonperfusion, and occlusion of retinal veins.

**Figure 3:**
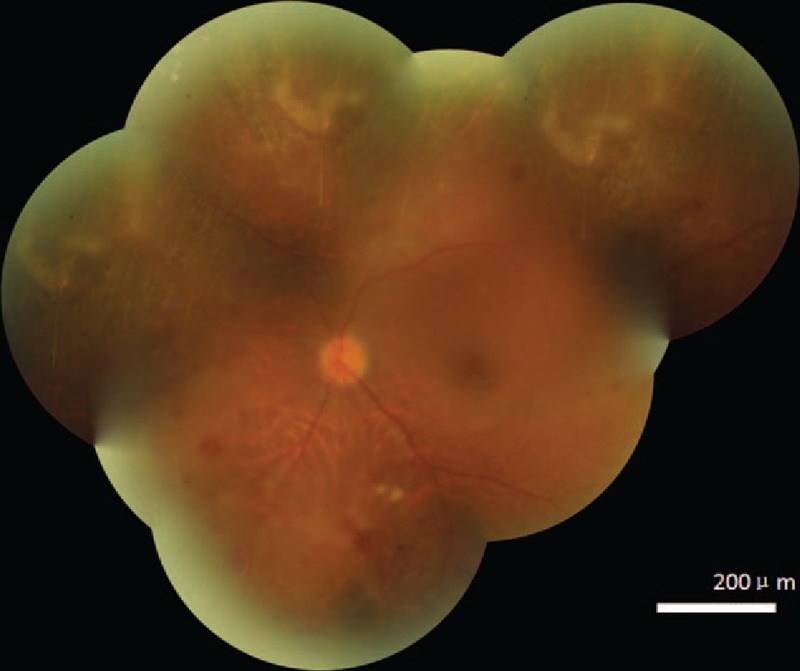
The cytomegalovirus retinitis and the perivascular infiltration regressed.

A 44-year-old male patient, who had been diagnosed with acute lymphosarcoma leukemia (ALL) and was receiving immunosuppressive therapy as a part of allogeneic HSCT, complained of an abrupt blurring of vision in his left eye (light perception) with normal eyeball movements and normal bulbar and palpebral conjunctiva. HIV testing was conducted twice in this patient, in both cases with negative results. His right eye was normal. However, his left eye showed the characteristics of CMV retinitis when viewed with a fundus camera (Fig. 1). Fluorescein angiography revealed retinal leakage, nonperfusion, and occlusive retinal vessels (Fig. 2). The concentration of the CMV DNA load in the aqueous humor was 2.24E + 05 copy/mL as assayed by the quantitative CMV DNA Polymerase chain reaction test. Therefore, a diagnosis of CMV retinitis was made, and his immunosuppressive therapy was discontinued. The patient refused to undergo a session of laser photocoagulation and oral valganciclovir. Instead, he was treated with ganciclovir 2 mg/mL (0.2 mL, 400 μg) intravitreal (IV) injection at weekly intervals for 4 weeks; 3 months later, his vision had improved (5/20) and the load of CMV DNA in the aqueous humor declined to 5.12E + 03 copy/mL. The CMV retinitis and perivascular of retina infiltration regressed (Fig. 3). The patient provided informed consent for this study.

## 3 Discussion

CMV retinitis is a common opportunistic infection in immunocompromised patients, usually in HIV patients who have low CD4+ T cells counts.^[7]^ In our immunosuppressed ALL patient, the characteristic lesions of fundus and laboratory assay of aqueous humor confirmed that he had developed CMV retinitis and in his case; this was not secondary to HIV.

Bone marrow transplantation usually requires high-dose immunosuppressant therapy, which may impair immunity and make the patient susceptible to CMV infection including ocular, even in patients not infected with HIV.^[8]^ One previous report claimed that about 20% to 35% of allogeneic HSCT recipients may develop CMV infection if they do not receive any antiviral prophylaxis.^[9]^ It is rather common that the retina is affected in acute leukemia, with about 70% of patients experiencing retinal symptoms,^[10]^ but it is very rare for an ALL patient undergoing allogeneic HSCT to suffer CMV disease. Since there were clear signs of lesions in the retina in the fundoscopic and fluorescein angiographic evaluation, the pathology of this case of CMV retinitis seemed to involve vascular occlusion, hemorrhage, and exudates. We speculate that the endothelial cells of the retina capillary wall are the crucial tissue being invaded by viral particles. In the clinic, the common treatment for CMV retinitis is laser photocoagulation combined with either oral or IV administration of antiviral drugs such as ganciclovir.^[11,12]^ However, the treatment outcomes for CMV retinitis tend to be poor and prone to relapse.^[13]^ In this case, we assayed the aqueous humor for CMV, which increased the reliability of the diagnosis. In addition, the concentration of CMV DNA load in the aqueous humor declined to 5.12E + 03 copy/mL, and the patient's vision improved. We propose that the concentration of CMV DNA load in the aqueous humor can be used to confirm the diagnosis and will subsequently be advantageous in selecting the appropriate treatment for secondary CMV retinitis in ALL patients receiving immunosuppressive therapy as a component of their allogeneic HSCT. It is noteworthy that the CMV DNA load in aqueous humor is normal value in the patients with CMV infection undergoing HSCT but having not retinitis, rather something like hepatitis and encephalitis, or in the most commonly seen cases such as pneumonia or gastrointestinal tract infection. In addition, there were some risk factors for secondary CMV retinitis such as transplantation, the use of high-dose corticosteroids, and graft-versus-host disease.^[14–16]^

Furthermore, ophthalmologists should be aware that all patients who are being treated with immunosuppressive drugs may potentially have an elevated risk of suffering a CMV infection.
